# An observational study of the prevalence of metabolic syndrome in treatment-experienced people living with HIV in Singapore

**DOI:** 10.1371/journal.pone.0252320

**Published:** 2021-06-02

**Authors:** Li Wei Ang, Oon Tek Ng, Irving Charles Boudville, Yee Sin Leo, Chen Seong Wong

**Affiliations:** 1 National Public Health and Epidemiology Unit, National Centre for Infectious Diseases, Singapore; 2 Department of Infectious Diseases, National Centre for Infectious Diseases, Singapore; 3 Department of Infectious Diseases, Tan Tock Seng Hospital, Singapore; 4 Lee Kong Chian School of Medicine, Nanyang Technological University, Singapore; 5 Saw Swee Hock School of Public Health, National University of Singapore, Singapore; 6 Yong Loo Lin School of Medicine, National University of Singapore, Singapore; Harvard Medical School, UNITED STATES

## Abstract

**Background:**

While the use of combination antiretroviral therapy (cART) has conferred significant reduction in morbidity and mortality, there are growing concerns about the metabolic complications of antiretroviral regimens in HIV-infected patients. The aim of this study was to estimate the prevalence of metabolic syndrome (MetS) among people living with HIV (PLHIV) in Singapore.

**Methods:**

We conducted a retrospective study using the clinical database maintained by the Clinical HIV Programme at the National Centre for Infectious Diseases, Singapore. Treatment-experienced PLHIV on follow-up during 2015–2017 were included. MetS was defined as having three or more of the following five abnormalities: hypertriglyceridemia, HDL hypocholesterolemia, hypertension, obesity, and diabetes.

**Results:**

A total of 2,231 PLHIV were included in this study. 93.9% were men, and the median age at latest follow-up was 48 years. The median duration of HIV infection and duration of exposure to cART was 6.8 years and 5.7 years, respectively. All had been exposed to nucleoside reverse transcriptase inhibitors (NRTIs) as the first line of treatment, 93.9% to non-NRTIs, 28.6% to protease inhibitors (PIs) and 12.8% to integrase strand transfer inhibitors. The most common metabolic abnormality among PLHIV was HDL hypocholesterolemia (60.2%) followed by hypertriglyceridemia (45.5%). Of all the 2,231 individuals, 68.8% had at least one component of MetS. The overall prevalence of MetS was 23.6% (95% confidence interval 21.9%–25.4%). Of the 526 with MetS, the most common combination was HDL hypocholesterolemia, hypertriglyceridemia and hypertension (51.0%), followed by HDL hypocholesterolemia, hypertriglyceridemia, hypertension and diabetes (25.1%). Compared with PLHIV without MetS, a significantly higher proportion of those with MetS were ever on protease inhibitors (33.5% vs. 27.1%).

**Conclusion:**

MetS is common in PLHIV. In view of the progressive aging of HIV-infected population and long-term use of cART, regular monitoring for metabolic abnormalities, surveillance of drug effects and behavioural interventions are needed to optimize management and prevention of metabolic disorders in PLHIV.

## Introduction

Metabolic syndrome (MetS), a cluster of interrelated cardio-metabolic abnormalities, is associated with increased risk of cardiovascular events and deaths [1,2]. HIV infection in itself may predispose people living with HIV (PLHIV) to the pathophysiological mechanisms in the development of MetS [3,4]. HIV infection is known to cause derangements in lipid metabolism through a variety of mechanisms, particularly the combination of elevated triglycerides and reduced high-density lipoprotein (HDL) [3]. While the advent of combination antiretroviral therapy (cART) has led to significant improvements in prognosis and life expectancy of HIV-infected individuals, numerous physical and metabolic alterations have been ascribed to cumulative exposure to antiretroviral regimens [5–8]. In particular, the HIV protease inhibitor (PI) class have been implicated in the induction or acceleration of metabolic complications such as lipodystrophy and dyslipidemia, and may be associated with atherosclerosis and insulin resistance [9–14]. The increased risk of myocardial infarction associated with use of PIs has also been described, especially in PLHIV with longer exposure to treatment [6,15–17]. This highlights the increasing need for effective preventive and clinical management of HIV patients who are exposed to long-term metabolic toxicities of cART [18].

Based on a meta-analysis of 65 studies across five continents comprising over 55,000 HIV-infected participants aged 17–73 years, the prevalence of MetS according to various definitions ranged from 16.7% (Adult Treatment Panel [ATP] III-2001) to 31.3% (European Group for the Study of Insulin Resistance) [19]. A meta-analysis of MetS among PLHIV in developing countries found that the mean prevalence was 21.5% in the Asian region (India and Thailand) [20]. However, studies on the prevalence of MetS among PLHIV and the association with specific antiretroviral drug classes are relatively lacking in Asian countries [21].

The first case of HIV in Singapore was diagnosed in 1985. As at the end of 2019, there had been 8,618 Singapore residents with HIV infection, of whom 2,097 had died [22]. The utilization of antiretroviral treatment in Singapore is predicated on the availability of drugs, and consisted of single antiretroviral use before July 1995, single or dual agent-regimens from July 1995 to June 1996, and single, dual or multiple agents from July 1996 onwards [23,24]. A majority (97%) of newly diagnosed HIV infections was acquired through sexual transmission, with a preponderance of men (male-to-female ratio of 9:1 or higher) [22]. An increasing proportion of newly diagnosed cases aged 50 years and older has been observed over the years [25,26]. This has led to additional challenges in the clinical management of PLHIV, since older persons tend to have more comorbidities, and pre-existing cardiovascular, hepatic, and metabolic complications are often exacerbated by HIV infection and MetS as well as other adverse effects of cART [27].

There is a growing need for long-term clinical management of co-morbid chronic medical conditions among PLHIV along with their HIV infection–phenomena described as multi-morbidity and polypharmacy. Notably, the cardiovascular risk associated with the cluster of abnormalities defining MetS has been found to be greater than the risk associated with individual components of MetS, which could indicate synergistic effects or interactions between the five components [1]. Thus it is important to have a good understanding of the prevalence and presentation of MetS among PLHIV on cART, as drug effects and interactions that increase the risk of cardiovascular disease are known to be more prevalent in this population [28]. In this context, we estimated the prevalence of MetS as well as the components defining the syndrome among treatment-experienced PLHIV.

## Materials and methods

### Study design

We conducted a retrospective study using the records of PLHIV who were on follow-up under the Clinical HIV Programme at the National Centre for Infectious Diseases (NCID), which is the major referral centre for HIV care in Singapore. A clinical database is maintained under the programme, which collects detailed demographic data, virologic and immunologic parameters, information on co-infections and opportunistic infections, antiretroviral therapy and monitoring, and test results from routine biochemical investigations performed in the course of HIV care.

Individuals included in this retrospective study were Singapore residents diagnosed with HIV at 18 years or older who had ever received cART at the major referral centre for HIV care between 2006 and 2017. We confined the study sample to those with data on at least one of the five components of MetS from 2015 to 2017, as they were deemed to be on follow-up during this three-year period. We then excluded those without measurements on at least three of the five MetS components between 2006 and 2017. For example, if an individual had data on one of the five components of MetS from 2015 to 2017, but did not have data on at least two other MetS components between 2006 and 2014, he or she would be excluded from the study.

### Definition of metabolic syndrome

For our study, we considered a modified National Cholesterol Education Program ATP III definition of MetS [29] as used in the Data Collection on Adverse Events of Anti-HIV Drugs (DAD) study [30]. Abnormalities of MetS components were classified according to the clinical practice guidelines of the Singapore Ministry of Health (MOH) [31]. For the definition of MetS in our study, we used body mass index (BMI) as a surrogate for waist circumference, similar to two studies where measurement of waist circumference was not available [30,32]. MetS was defined as the presence of three or more of the following five abnormalities: hypertriglyceridemia (triglyceride level ≥150 mg/dL or ≥1.7 mmol/L), HDL hypocholesterolemia (HDL-cholesterol level <1.0 mmol/L or <40 mg/dL), hypertension (systolic blood pressure [SBP] ≥140 mmHg or diastolic blood pressure [DBP] ≥90 mmHg), obesity (BMI ≥30 kg/m^2^), and diabetes mellitus (fasting glucose ≥126 mg/dL or ≥7.0 mmol/L). Individuals met the criteria for hypertriglyceridemia, hyperlipidemia, hypertension and/or diabetes if they were on specific treatment, had been diagnosed with these metabolic abnormalities, or had two or more consecutive abnormal laboratory results or measurements.

We assumed that abnormalities in all these five MetS components were irreversible; once an individual had met any of the criteria, he or she would be deemed to always meet the criteria. For this study, we followed the recommendation that the prevalence of MetS in cohort studies should be based on two consecutive measurements of the laboratory components in order to minimize bias due to missing or highly variable measurements [30].

### Laboratory investigations

HIV infection was confirmed by western blot assay upon a positive result from a fourth generation chemiluminescent microparticle immunoassay, as per the local diagnostic algorithm. HDL-cholesterol, glucose and triglyceride levels were determined from blood specimens of HIV-infected patients after eight hours of fasting. Hepatitis B virus (HBV) infection was ascertained based on seropositivity for hepatitis B antigen surface (HBsAg) using electrochemiluminescence immunoassay (ECLIA), and hepatitis C virus infection (HCV) was based on seropositivity for the anti-HCV antibody (anti HCV) test using enzyme-linked immunoassay (ELISA) or detectable HCV with real-time polymerase chain reaction (PCR) [33].

### Statistical analysis

The prevalence of MetS was calculated along with 95% confidence interval (CI) for binomial proportions using the Wilson’s method. We compared the epidemiological and clinical characteristics among PLHIV with and without MetS using chi-square test or Fisher’s exact test for categorical variables where appropriate, and Mann-Whitney test for continuous variables.

The cumulative duration of exposure was calculated for PLHIV who had ever received treatment from each drug class. For PLHIV who were still on cART over the study period, we used 31 December 2017 as the end date to calculate the cumulative duration of exposure. For each drug class, we followed the grouping of duration in a study on prevalence of MetS among HIV-infected Taiwanese patients [21]: <12 months, 12–35 months, 36–71 months and ≥72 months.

All *p* values reported were 2-sided and statistical significance was taken as *p* < 0.05. Statistical analyses were performed using the IBM SPSS Statistics for Windows version 24.0 (IBM Corp., Armonk, NY, USA) and R version 3.6.2 (R Foundation for Statistical Computing, Vienna, Austria).

### Ethical considerations

Ethics approval for use of the clinical database was obtained from the Singapore National Healthcare Group Domain Specific Review Board (NHG DSRB reference number 2012/00438). Informed consent was not obtained as the clinical data collected was used as part of the care management of HIV patients. All data analyzed for the study were anonymized.

## Results

Among the 3,327 HIV-infected patients seen under the Clinical HIV Programme from 2006 to 2017, 1,096 were excluded from the analysis for the following reasons: 12 were aged below 18 years at time of HIV diagnosis, 158 had not been on cART, 572 did not have data on at least one of the five MetS components between 2015 and 2017, and 354 did not have measurements on at least three of the five MetS components between 2006 and 2017. A total of 2,231 PLHIV were included in the study.

HIV-infected patients aged 25–54 years at HIV diagnosis comprised 76.0% of the study sample (Table 1). The median age at HIV diagnosis was 41 years (interquartile range [IQR] 32–50). As of end 2017, 43.2% of the PLHIV were of age 50 years or older on their latest follow-up for HIV care, and the median age was 48 years (IQR 39–57). 93.9% were men and 78.0% were Chinese. The main mode of HIV transmission was via sexual exposure (96.5%). The median duration of HIV infection was 82 months (6.8 years). More than two-thirds (64.9%) did not have prior AIDS diagnosis, and 46.9% had CD4 ≤200 cells/mm^3^ at the time of HIV diagnosis. About 35.5% of the individuals did not have HIV viral load measurements within 6 months of HIV diagnosis, while among 1,439 with known HIV viral load, 1,369 (95.1%) were not virologically suppressed (>200 copies/mL). The majority had been tested negative for HBV (87.9%) and HCV (91.7%).

**Table 1:** Epidemiological and clinical characteristics of people living with HIV on follow-up between 2015 and 2017 by status of metabolic syndrome.

Characteristic	All	MetS	No MetS	*P* value
	(n = 2,231)	(n = 526)	(n = 1,705)	
Age at HIV diagnosis, median (IQR)	41 (32–50)	49 (41–56)	38 (30–47)	<0.0005
Age at HIV diagnosis, n (%)				<0.0005
18–24	187 (8.4)	10 (1.9)	177 (10.4)	
25–34	517 (23.2)	45 (8.6)	472 (27.7)	
35–44	642 (28.8)	131 (24.9)	511 (30.0)	
45–54	536 (24.0)	187 (35.6)	349 (20.5)	
55–64	288 (12.9)	121 (23.0)	167 (9.8)	
65+	61 (2.7)	32 (6.1)	29 (1.7)	
Age at latest follow-up, median (IQR)	48 (39–57)	56 (48–63)	44 (36–54)	<0.0005
Age at latest follow-up, n (%)				<0.0005
18–29	196 (8.8)	6 (1.1)	190 (11.1)	
30–39	442 (19.8)	35 (6.7)	407 (23.9)	
40–49	629 (28.2)	110 (20.9)	519 (30.4)	
50–59	566 (25.4)	185 (35.2)	381 (22.3)	
60–69	320 (14.3)	145 (27.6)	175 (10.3)	
70+	78 (3.5)	45 (8.6)	33 (1.9)	
Gender, n (%)				0.835
Male	2,095 (93.9)	493 (93.7)	1,602 (94.0)	
Female	136 (6.1)	33 (6.3)	103 (6.0)	
Ethnic group, n (%)				<0.0005
Chinese	1,741 (78.0)	437 (83.1)	1304 (76.5)	
Malay	316 (14.2)	45 (8.6)	271 (15.9)	
Indian	101 (4.5)	27 (5.1)	74 (4.3)	
Others	73 (3.3)	17 (3.2)	56 (3.3)	
HIV transmission risk group, n (%)				<0.0005
Homosexual/bisexual	1,074 (48.1)	131 (24.9)	943 (55.3)	
Heterosexual	1,008 (45.2)	363 (69.0)	645 (37.8)	
IDU	10 (0.4)	2 (0.4)	8 (0.5)	
IDU and sexual	70 (3.1)	11 (2.1)	59 (3.5)	
Others and unknown	69 (3.1)	19 (3.6)	50 (2.9)	
Duration since HIV diagnosis (months), median (IQR)	82 (49–115)	93 (56–116)	69 (35–101)	<0.0005
Period of HIV diagnosis, n (%)				<0.0005
2006–2008	649 (29.1)	216 (41.1)	433 (25.4)	
2009–2011	634 (28.4)	153 (29.1)	481 (28.2)	
2012–2014	565 (25.3)	102 (19.4)	463 (27.2)	
2015–2017	383 (17.2)	55 (10.5)	328 (19.2)	
Prior AIDS diagnosis, n (%)				<0.0005
No	1,449 (64.9)	307 (58.4)	1142 (67.0)	
Yes	782 (35.1)	219 (41.6)	563 (33.0)	
CD4 (cells/mm^**3**^) at HIV diagnosis^§^, median (IQR), n = 2,124	205 (46–360)	105 (27–286)	236 (61–378)	<0.0005
CD4 (cells/mm^**3**^) at HIV diagnosis^§^, n (%)				<0.0005
≤200	1,047 (46.9)	327 (62.2)	720 (42.2)	
201–350	525 (23.5)	99 (18.8)	426 (25.0)	
>350	552 (24.7)	84 (16.0)	468 (27.4)	
Not available	107 (4.8)	16 (3.0)	91 (5.3)	
Plasma HIV-1 RNA (copies/mL) at diagnosis^§^, median (IQR), n = 1,439	5.0 (4.4–5.6)	5.2 (4.5–5.8)	4.9 (4.3–5.5)	0.789
Plasma HIV-1 RNA (copies/mL) at diagnosis^§^, n (%)				<0.0005
>200	1,369 (61.4)	255 (48.5)	1114 (65.3)	
≤200	70 (3.1)	22 (4.2)	48 (2.8)	
Not available	792 (35.5)	249 (47.3)	543 (31.8)	
Latest measurements in 2015–2017, median (IQR)				
Triglycerides (mmol/L), n = 2,212	1.4 (0.9–2.1)	2.1 (1.4–2.8)	1.2 (0.9–1.8)	<0.0005
Total cholesterol (mmol/L), n = 2,212	4.9 (4.3–5.5)	5.0 (4.3–5.7)	4.9 (4.3–5.5)	0.008
HDL (mmol/L), n = 2,212	1.2 (1.0–1.4)	1.0 (0.9–1.3)	1.2 (1.0–1.4)	<0.0005
LDL (mmol/L), n = 2,158	3.0 (2.4–3.5)	2.9 (2.3–3.4)	3.0 (2.5–3.5)	0.020
Fasting glucose (mmol/L), n = 1,807	5.2 (4.8–5.6)	5.7 (5.1–7.0)	5.1 (4.8–5.5)	<0.0005
SBP (mmHg), n = 1,594	123 (114–134)	132 (121–142)	121 (112–131)	<0.0005
DBP (mmHg), n = 1594	70 (64–78)	76 (69–84)	69 (63–76)	<0.0005
BMI (kg/m^2^), n = 1084	22.6 (20.3–25.0)	24.9 (22.4–28.4)	22.0 (19.9–23.9)	<0.0005
Ever had HBV coinfection, n (%)				0.005
No	1,961 (87.9)	482 (91.6)	1479 (86.7)	
Yes	161 (7.2)	22 (4.2)	139 (8.2)	
Unknown	109 (4.9)	22 (4.2)	87 (5.1)	
Ever had HCV coinfection, n (%)				0.067
No	2,046 (91.7)	492 (93.5)	1554 (91.1)	
Yes	126 (5.6)	19 (3.6)	107 (6.3)	
Unknown	59 (2.6)	15 (2.9)	44 (2.6)	
Exposure to cART classes, n (%)				
NRTIs	2,231 (100.0)	526 (100.0)	1,705 (100.0)	-
Tenofovir-containing	1,683 (75.4)	347 (66.0)	1336 (78.4)	<0.0005
Abacavir -containing	1,244 (55.8)	319 (60.6)	925 (54.3)	0.010
NNRTIs	2094 (93.9)	503 (95.6)	1,591 (93.3)	0.061
PIs	638 (28.6)	176 (33.5)	462 (27.1)	0.006
INSTIs	286 (12.8)	68 (12.9)	218 (12.8)	0.941
Duration of cART (months), median (IQR)^╪^				
All drug classes	67.7 (38.9–98.6)	86.0 (53.9–114.2)	62.0 (36.1–89.9)	<0.0005
NRTIs	66.5 (38.2–98.0)	85.2 (52.2–113.4)	60.4 (35.3–89.7)	<0.0005
Tenofovir-containing, n = 1,683	39.6 (16.2–65.2)	40.2 (14.2–68.6)	39.5 (16.5–65.0)	0.923
Abacavir -containing, n = 1,244	20.5 (10.5–35.9)	23.5 (12.0–40.7)	19.6 (10.1–33.8)	0.004
NNRTIs, n = 2,094	54.3 (24.0–88.5)	68.5 (27.8–108.7)	51.3 (22.9–83.7)	<0.0005
PIs, n = 638	37.3 (12.5–70.1)	42.1 (15.2–75.6)	34.4 (12.2–66.5)	0.096
INSTIs, n = 286	11.9 (4.2–34.3)	11.1 (4.6–35.8)	12.0 (4.1–34.1)	0.694
Ever used recreational or illicit drugs†, n (%)				0.001
No	424 (19.0)	92 (17.5)	332 (19.5)	
Yes	220 (9.9)	31 (5.9)	189 (11.1)	
Unknown	1,587 (71.1)	403 (76.6)	1184 (69.4)	
Current smoking, n (%)				0.236
No	270 (12.1)	73 (13.9)	197 (11.6)	
Yes	223 (10.0)	57 (10.8)	166 (9.7)	
Unknown	1,738 (77.9)	396 (75.3)	1,342 (78.7)	
Alcohol consumption, n (%)				0.001
Never	693 (31.1)	191 (36.3)	502 (29.4)	
Sometimes	570 (25.5)	111 (21.1)	459 (26.9)	
Daily	62 (2.8)	21 (4.0)	41 (2.4)	
Drinks but frequency unknown	63 (2.8)	20 (3.8)	43 (2.5)	
Ex-drinker	132 (5.9)	33 (6.3)	99 (5.8)	
Unknown	711 (31.9)	150 (28.5)	561 (32.9)	

Sample size, n = 2,231, except where indicated. Data are expressed as n (%) or median (interquartile range).

^§^ Within ± 6 months of HIV diagnosis.

^**╪**^ The duration of patients not on the specific drug class was treated as missing data, and excluded from computation.

† Includes ecstasy, ’poppers’, viagra, amphetamines, cannabis, heroin, cocaine, barbiturates/ benzodiazepines, opium, psychedelic mushrooms, solvents, LSD (Lysergic Acid Diethylamide).

cART, combination antiretroviral therapy; DBP, diastolic blood pressure; HDL, high-density lipoprotein; INSTIs, integrase strand transfer inhibitors; IQR, interquartile range; LDL, low-density lipoprotein; MetS, metabolic syndrome; NNRTIs, non-nucleoside reverse transcriptase inhibitors; NRTIs, nucleoside reverse transcriptase inhibitors; PIs, protease inhibitors; SBP, systolic blood pressure.

The median duration of exposure to cART was 68 months (5.7 years) (Table 1). All the PLHIV had been exposed to nucleoside reverse transcriptase inhibitors (NRTIs) as the first line of treatment. 28.6% had a history of exposure to PIs and 12.8% to integrase strand transfer inhibitors (INSTs). The most common combination of cART was two NRTIs plus one non-nucleoside reverse-transcriptase inhibitor (NNRTI), followed by two NRTIs plus one PI (S1 Table). The most common antiretroviral agent by drug class was tenofovir from NRTIs, efavirenz from NNRTs, ritonavir from PIs and raltegravir from INSTIs.

The most common metabolic abnormality was HDL hypocholesterolemia (60.2%) followed by hypertriglyceridemia (45.5%) (Table 2). Of all the 2,231 individuals, 68.8% had at least one metabolic abnormality, and of these 1,536 individuals, 25.7% had one component, 40.0% had two components, 23.2% had three components, 10.0% had four components and 1.0% had all five MetS components. The overall prevalence of MetS was 23.6% (95% confidence interval 21.9%–25.4%). Of these 526 individuals with MetS, all had HDL hypocholesterolemia, 91.3% had hypertriglyceridemia and 90.7% had hypertension.

**Table 2:** Proportion with individual metabolic abnormalities among people living with HIV and those with metabolic syndrome on follow-up between 2015 and 2017.

Metabolic abnormality, n (%)	All (n = 2,231)	PLHIV with MetS (n = 526)
HDL hypocholesterolemia	1,344 (60.2%)	526 (100.0%)
Hypertriglyceridemia	1,016 (45.5%)	480 (91.3%)
Hypertension	712 (31.9%)	477 (90.7%)
Diabetes	258 (11.6%)	227 (43.2%)
Obesity	57 (2.6%)	52 (9.9%)

MetS, metabolic syndrome, PLHIV, people living with HIV.

**Fig 1:**
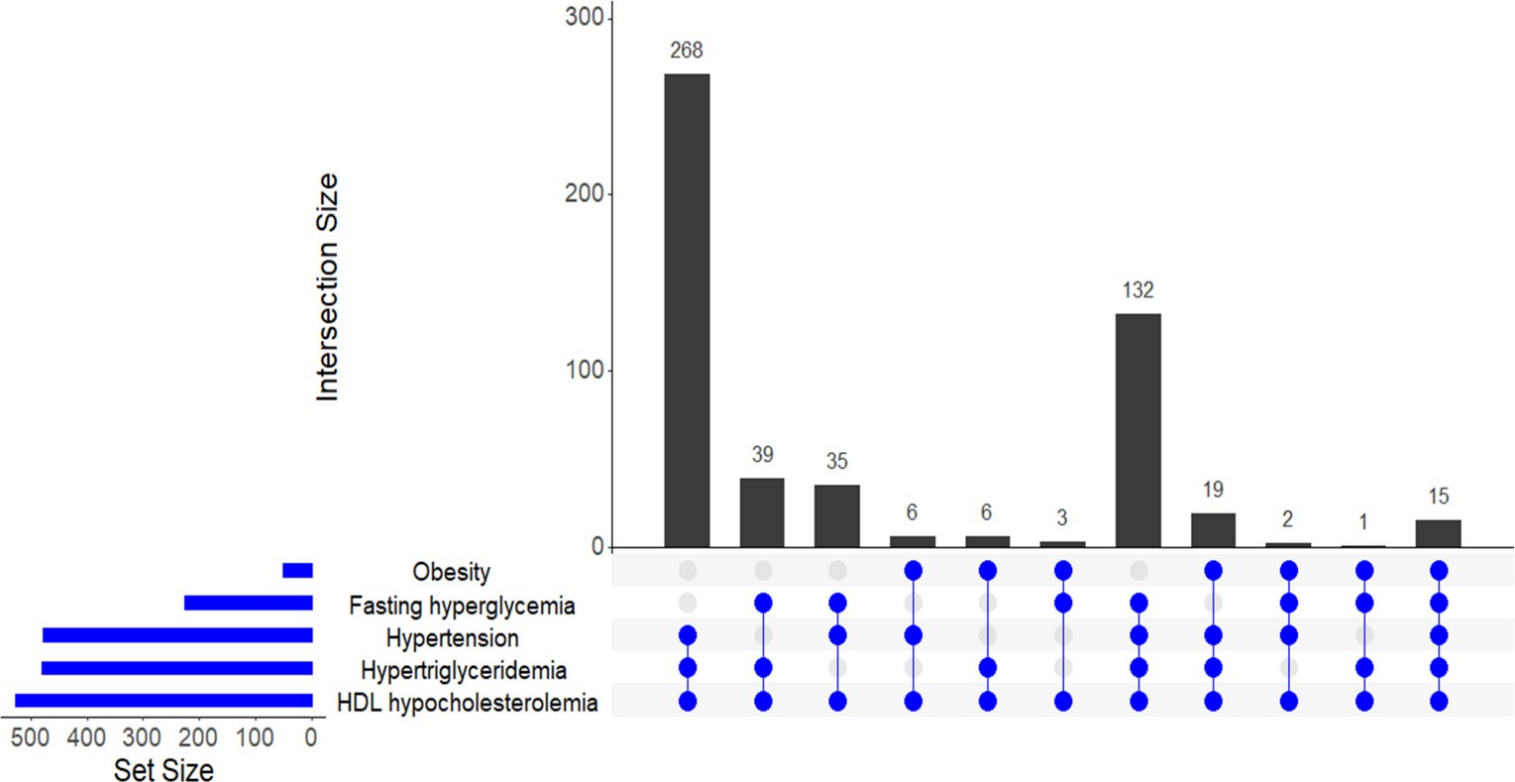
Frequency of combinations of metabolic abnormalities among people living with HIV with metabolic syndrome on follow-up between 2015 and 2017’.

The distribution of the various combinations of metabolic abnormalities in PLHIV with MetS is shown in Fig 1. The most common combination was HDL hypocholesterolemia, hypertriglyceridemia and hypertension (51.0%), followed by the combination of HDL hypocholesterolemia, hypertriglyceridemia, hypertension and diabetes (25.1%). About 67.9% of PLHIV with MetS had three metabolic abnormalities, 29.3% had four metabolic abnormalities and 2.8% had all the five metabolic abnormalities.

**Fig 2:**
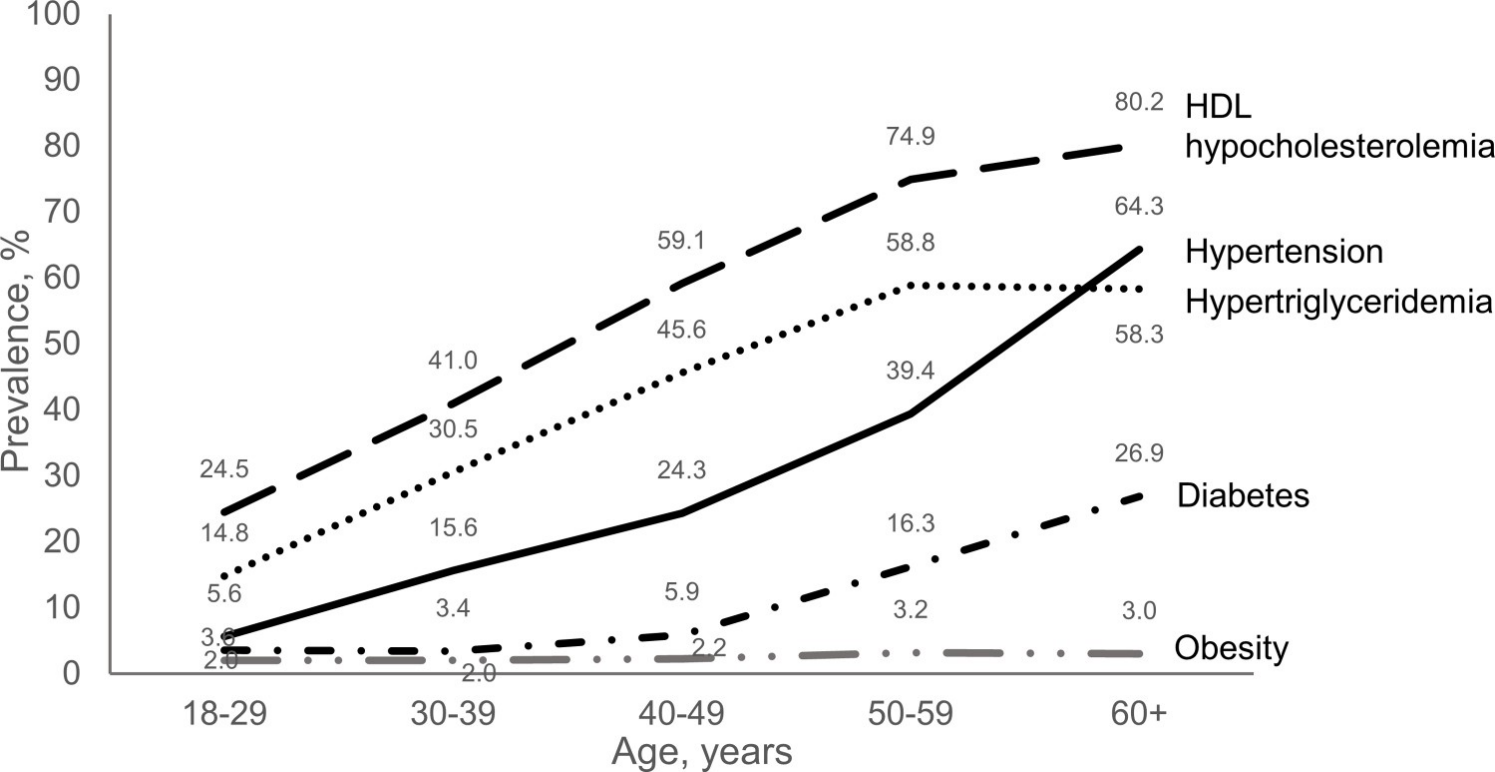
Age-specific prevalence of metabolic abnormalities among people living with HIV on follow-up between 2015 and 2017.

The prevalence of MetS increased from 3.1% in young adults aged 18–29 years to 47.7% in elderly aged 60 years or older (test for trend, *p*<0.0005). A breakdown by individual metabolic abnormality revealed that the prevalence of HDL hypocholesterolemia, hypertriglyceridemia, hypertension and diabetes increased by age group (test for trend, *p*<0.0005) whereas no linear trend was observed for obesity (test for trend, *p* = 0.224) (Fig 2).

Compared with PLHIV without MetS, a significantly higher proportion of those with MetS were aged 45 years or older at HIV diagnosis (64.6% vs. 32.0%), aged 50 years or older at latest follow-up (71.3% vs. 34.5%), Chinese (83.1% vs. 76.5%), infected with HIV via heterosexual mode of transmission (69.0% vs. 37.8%), diagnosed in 2006–2008 (41.1% vs. 25.4%) and had prior AIDS diagnosis (41.6% vs. 33.0%) (all *p*<0.0005) (Table 1). The duration since HIV diagnosis was longer in PLHIV with MetS compared with those without MetS (median duration was 93 vs. 69 months). PLHIV with MetS were more likely to have higher levels of triglycerides, total cholesterol, fasting glucose, SBP, DBP and BMI, and lower levels of HDL-cholesterol and LDL-cholesterol.

**Fig 3:**
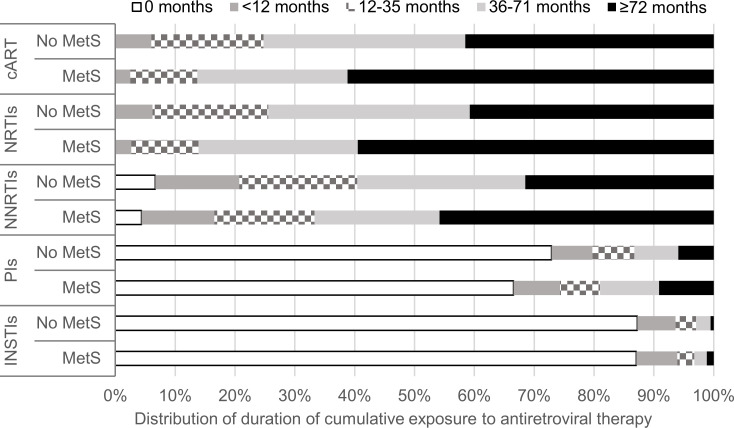
Distribution of duration of cumulative exposure to combination antiretroviral therapy by status of metabolic syndrome and drug class among people living with HIV on follow-up between 2015 and 2017. cART, combination antiretroviral therapy; INSTIs, integrase strand transfer inhibitors; MetS, metabolic syndrome; NNRTIs, non-nucleoside reverse transcriptase inhibitors; NRTIs, nucleoside reverse transcriptase inhibitors; PIs, protease inhibitors.

Compared with PLHIV without MetS, a significantly higher proportion of those with MetS had exposure to PIs (33.5% vs. 27.1%) (Table 1). The cumulative duration of exposure to cART, NRTIs and NNRTIs for PLHIV who had ever been on these antiretroviral regimens was significantly longer in the group with MetS than in those without MetS. Compared with PLHIV without MetS, significantly higher proportions had cumulative duration of exposure ≥72 months among those with MetS who were ever on cART (61.2% vs. 41.5%), NRTI (59.5% vs. 40.8%), NNRTIs (45.8% vs. 31.5%) and PIs (9.1% vs. 5.9%) (Fig 3).

## Discussion

We estimated that nearly one-quarter of treatment-experienced PLHIV on follow-up for HIV care in 2015–2017 had MetS. The prevalence of MetS in our study was similar to that reported in other countries [19,20], although there were differences in the criteria for defining the MetS components, duration since HIV diagnosis, proportion receiving cART and duration of exposure to various drug classes, prevalence of cardiovascular risk factors and comorbidities, and the heterogeneity of HIV positive populations.

The prevalence of metabolic abnormality for each component of MetS exhibits substantial variability within and across HIV positive populations [34]. The most common components of MetS in our study were HDL hypocholesterolemia and hypertriglyceridemia, similar to what had been observed in the DAD study of PLHIV in Europe, Australia and the United States [30]. Obesity was the MetS component with the lowest prevalence among PLHIV (2.6%) in our study, which was also the case in the DAD study [30].

There have not been any recent published studies on prevalence of MetS in Singapore’s general population. Some studies have found that the prevalence of MetS was higher in PLHIV than the general population in Taiwan [21], Italy [35] and Poland [36], while a study in an urban, midwestern US outpatient population did not find any significant difference between the two groups [37]. In another US study, PLHIV were less likely to have MetS than the general population when no additional adjustment was made for BMI in a multivariable analysis, regardless of whether they had received cART, and one explanation was that hypertriglyceridemia and HDL hypocholesterolemia were more likely in PLHIV compared with the general population [38].

The prevalence of MetS was higher in older PLHIV and in those of Chinese ethnicity. Older age had been identified as an independent risk factor associated with MetS among HIV-infected patients in several studies [34,39–43]. While higher prevalence of MetS was seen in Chinese PLHIV, they were of significantly older age compared with Malays (average age was 48 years vs. 42 years).

Exposure to cART has been reported to be associated with a number of comorbidities, and the long-term effects of individual antiretroviral agents are still being discovered [28]. In our study, the proportion of PLHIV with MetS who had ever been exposed to PIs was higher than those without MetS. The proportion with cumulative duration of exposure ≥72 months on PIs was also higher in those with MetS. A meta-analysis found that the use of PIs was associated with the development of MetS [44]. As all individuals in our study had received NRTIs, we were unable to elucidate whether the use of this drug class was associated with MetS in PLHIV.

This study included new findings pertaining to INSTIs, which are the latest class of antiretroviral drugs incorporated into the treatment recommendations for both treatment-naïve as well as treatment-experienced individuals with HIV [45]. The median duration of exposure for PLHIV who had ever received INSTIs was shorter than for those on PIs. Data from a large North American HIV cohort suggested that initiation of first cART regimens with INSTIs versus NNRTIs may confer an increased risk of incident diabetes, likely mediated through weight gain [46]. Studies have found weight gain following a switch to INSTIs, with more pronounced increases in HIV-positive women and with the use of dolutegravir [47–52]. It remains to be seen whether prolonged exposure to INSTIs would increase the risk of metabolic disorders [53]. Further studies are required to evaluate this, especially with the increasing use of INSTIs in Singapore.

We acknowledge several limitations in this study. As our study population was confined to PLHIV seen at a single referral centre for HIV care, it may not be representative of HIV-positive individuals on follow-up at other healthcare institutions. However, the Clinical HIV Programme at NCID sees the largest number of PLHIV in Singapore and of 5,279 newly diagnosed cases of HIV infection notified to MOH between 2006 and 2017, 3,327 (63.0%) had sought HIV care at this centre at some time during the 12-year period. Hence our study provides a reasonable indication of the prevalence of MetS among PLHIV in Singapore. A substantial number of PLHIV did not have adequate data on MetS components, and had to be excluded from our analysis. There was a high proportion of missing data on current smoking and alcohol consumption. Information on medical history, use of medications, physical activity and dietary habits was not available in the clinical database. Having metabolic abnormalities may affect the choice of antiretroviral agents prescribed by the treating physician, especially if these were pre-existing conditions prior to HIV diagnosis.

In conclusion, MetS is common in PLHIV, and it is expected that the prevalence will increase with progressive ageing of the HIV positive population. Longer life expectancy of PLHIV and long-term use of cART have raised the concern of increased cardiovascular risk. Regular monitoring and assessment of metabolic components, surveillance of drug effects and behavioural interventions are needed to optimize management and prevention of metabolic disorders in PLHIV.

## Supporting information

S1 Table Frequency of combination of exposure to drug classes among treatment-experienced people living with HIV on follow-up at the national referral centre for HIV care in Singapore between 2015 and 2017. (DOCX)
