# Supplementary material for: An observational study of the prevalence of metabolic syndrome in treatment-experienced people living with HIV in Singapore
Source: PLoS One. 2021 Jun 2;16(6):e0252320. doi: 10.1371/journal.pone.0252320 (PMC8171957; doi:10.1371/journal.pone.0252320)
Supplement: S1 Table — (DOCX) [file pone.0252320.s001.docx]

**An observational study of the prevalence of metabolic syndrome in treatment-experienced people living with HIV in Singapore**

Li Wei Ang^1^, Oon Tek Ng^2,3,4^, Irving Charles Boudville^1^, Yee Sin Leo^2,3,4,5,6^, Chen Seong Wong^2,3,4^

^1^ National Public Health and Epidemiology Unit, National Centre for Infectious Diseases, Singapore

^2^ Department of Infectious Diseases, National Centre for Infectious Diseases, Singapore

^3^ Department of Infectious Diseases, Tan Tock Seng Hospital, Singapore

^4^ Lee Kong Chian School of Medicine, Nanyang Technological University, Singapore

^5^ Saw Swee Hock School of Public Health, National University of Singapore

^6^ Yong Loo Lin School of Medicine, National University of Singapore, Singapore

**Supplementary Table 1. Frequency of combination of exposure to drug classes among treatment-experienced people living with HIV on follow-up at the national referral centre for HIV care in Singapore between 2015 and 2017.**

| Combination of exposure to drug classes^╪^ | | | | No. | % |
| --- | --- | --- | --- | --- | --- |
| NRTIs | NNRTIs | PIs | INSTIs |  |  |
| X | X |  |  | 1,474 | 66.1 |
| X | X | X |  | 396 | 17.7 |
| X | X | X | X | 132 | 5.9 |
| X | X |  | X | 92 | 4.1 |
| X |  | X |  | 75 | 3.4 |
| X |  | X | X | 35 | 1.6 |
| X |  |  | X | 27 | 1.2 |

^╪^ The combination of drug classes treatment-experienced people living with HIV had ever been exposed to is in descending order of frequency.

INSTIs, integrase strand transfer inhibitors; NNRTIs, non-nucleoside reverse transcriptase inhibitors; NRTIs, nucleoside reverse transcriptase inhibitors; PIs, protease inhibitors
